# Mass intraoperative endothelial glycocalyx shedding affects postoperative systemic inflammation response

**DOI:** 10.1186/s12871-024-02459-z

**Published:** 2024-02-26

**Authors:** JiaWan Wang, Yan Wu

**Affiliations:** Department of Anesthesiology, Beijing Chaoyang Hospital Affiliated to Capital Medical University, Beijing, China

**Keywords:** Endothelial glycocalyx, Syndecan-1, Atrial natriuretic peptide, Systemic inflammation response syndrome

## Abstract

**Bacground:**

Off-pump coronary artery bypass graft (OPCABG) has a high incidence of postoperative systemic inflammation response syndrome (SIRS), and perioperative endothelial glycocalyx layer (EGL) disruption can be one of the predisposing factors. We hypothesized that EGL shedding happened earlier in OPCABG which can influence on postoperative SIRS, and sevoflurane might preserve EGL better than propofol.

**Methods:**

We randomly allocated 50 patients undergoing OPCABG to receive either sevoflurane-sufentanil or propofol-sufentanil anesthesia. Plasma syndecan-1, heparan sulfate (HS), atrial natriuretic peptide (ANP), IL-6, and cardiac troponin I (cTnI) were measured. Blood samples were collected at 6 timepoints: induction (T_1_), before grafting (T_2_), after grafting(T_3_), surgery done (T_4_), postoperative day1 (POD1,T_5_) and POD2 (T_6_). SIRS criteria and sequential organ failure assessment (SOFA) score were examined.

**Results:**

There were neither differences of syndecan-1, HS, IL-6 nor of SIRS criteria or SOFA score between the sevoflurane and propofol groups. All patients were pooled as a single group for further statistical analyses, plasma syndecan-1 (*P* < 0.001) and IL-6 (*P* < 0.001) increased significantly as a function of time; syndecan-1 increasing correlated significantly with the duration of coronary graft anastomosis (*r* = 0.329, *P* = 0.026). Syndecan-1(T_3_) correlated significantly with ANP(T_3_) (r = 0.0.354, *P* = 0.016) and IL-6 (T_5_) (*r* = 0.570, *P* < 0.001). The maximum value of IL-6 correlated significantly with SIRS (*r* = 0.378, *P* = 0.010), the maximum value of SOFA score (*r* = 0.399, *P* = 0.006) and ICU days (*r* = 0.306, *P* = 0.039). The maximum value of SOFA score correlated significantly with the occurrence of SIRS (*r* = 0.568, *P* < 0.001) and ICU days (*r* = 0.338, *P* = 0.022).

**Conclusions:**

OPCABG intraoperative early EGL shedding caused of grafts anastomosis greatly affected postoperative SIRS and SOFA score, sevoflurane did not clinically preserve EGL better.

**Trial registration:**

ChiCTR-IOR-17012535. Registered on 01/09/2017.

## Introduction

In major surgery especially cardiac operation, surgical incisions are highly traumatic accesses that impose extensive injury to tissues and can elicit an extended inflammatory response and severe postoperative immunosupression, which may result in poor prognosis from SIRS [1–3]; Compared to other kinds of surgery, the incidence of postoperative SIRS has a comparatively higher incidence rate in cardiac surgery including Off-Pump CABG although there is no cardiopulmonary bypass (CPB)and aortic clamping [4, 5]. Although SIRS has had a decreasing trend in recent decades, OPCABG has an approximate occurence of systemic inflammatory response and a similar endothelial response to conventional CABG [6–8]. EGL plays an important role in capillary endothelial structure and function. It is an integral luminal part of the vascular barrier composed of glycoproteins and proteoglycans [9, 10]. Luminal endothelial surface-bound EGL has various physiological functions, and plays a regulatory role in the adhesion of leukocytes and platelets as well as hemostasis and coagulation on the endothelial surface which can influence inflammation processes [11–13]. In cardiac surgery, EGL is damaged earlier and badly by various stimuli, such as endogenous ANP releasing, inflammation response, oxidative stress, and hypervolaemia which causes EGL shedding and an increase in leukocyte–endothelial interactions [14–16]. EGL shedding releases soluble ectodomains of syndecans act a distinct role in inflammation response, but on the other hand, the macromolecular substances delivered from EGL damage are an important source of damage-associated molecular patterns (DAMPs) which may in turn aggravate system inflammation process [17–19]. Overall, the deleterious impact of these pathophysiology on EGL could be evident by elevated plasma levels of glycocalyx components, mainly syndecan-1 and heparan sulfate, and soluble glycocalyx components could be a marker of system inflammation [20, 21].

Literatures report sevoflurane can preserve EGL in ischemia–reperfusion (I/R) injury, but large dose propofol might hurt EGL [22–24]. As OPCABG surgery probably has an earlier intraoperative EGL shedding, we hypothesized that the degradation products which can act as DAMPs may influence postoperative systemic inflammation response, and compared to propofol sevoflurane anesthesia may cause less EGL degradation and systemic inflammation response. Therefore, this clinical study aimed to compare the effects of sevoflurane-sufentanil or propofol-sufentanil anesthesia on endothelial glycocalyx degradation and systemic inflammation response in patients undergoing OPCAB surgery, and discuss the correlation between EGL degradation and perioperative SIRS.

## Materials and methods

This study was approved by the Medical Ethics Committee of Beijing ChaoYang Hospital Affiliated to Capial Medical University (approval number: 2017–8-23–49) and registered with the Chinese Clinical Trial Registry (http://www.chictr.org.cn; ChiCTR-IOR-17012535. Registered on 01/09/2017). Inclusion criteria for a patient were: written informed consent; elective OPCABG surgery; age over 40 years old; and American Society of Anesthesiologists (ASA) physical status of II–IV. Exclusion criteria were: body mass index > 35 kg.m^−2^; left ventricular ejection fraction < 35%; clinically relevant obstructive or restrictive lung diseases; pulmonary hypertension (systolic pulmonary arterial pressure > 50 mmHg); obvious coagulation disorders; severe pulmonary or systemic infections; chronic liver disease; chronic kidney disease; pre-operative use of steroids; and tobacco abuse, were not included in the study.

Preliminary trial showed that the concentration of serum syndecan-1 reached the highest level at POD1 in both groups and the mean difference between the propofol group and sevoflurane group was 13 ng.ml^−1^(δ = 13), while the standard deviations (SD) of the two groups were 15.5 vs 14.6. PASS 15 for sample size calculation showed that when statistical power was 0.8 (β = 0.20) and α was 0.05, bilateral testing required 23 cases for each group. Considering a 10% lost follow-up rate, 50 patients were enrolled and divided randomly into propofol group (P group, *n* = 25) or sevoflurane group (S group, *n* = 25) based on a list of random numbers generated by the random function in MS-Excel. The enrollment evaluation and assignment were carried out by dedicated persons who were not involved in surgery, anesthesia, specimen collection, data recording and analysis. The randomization codes were concealed in numbered and sealed envelopes. Early morning on the day of surgery the anesthetist revealed the treatment allocation for the patient by opening the next envelope and the anesthetist was not involved in later data processing. The surgeon and patient were not informed of whether it was sevoflurane inhalation or propofol intravenous anesthesia.

### Anesthesiological and perioperative procedure

Patients maintained their regular medication treatment for heart disease until the day of surgery and injection of morphine 8 mg as pre-operative medication. On arrival of operation room, patients were monitored with a 5-lead electrocardiogram, a saturation of pulse oximetry (SpO_2_), bispectral index (BIS)monitoring (BIS XP ®, Aspect Medical Systems, Newton, MA) and radial artery pressure (ART). A warming blanket and a warming infusion system were used for maintaining body temperature. Anesthesia was induced using i.v. etomidate 0.2 mg.kg^−1^, sufentanil 1.5 μg.kg^−1^, and rocuronium 0.9 mg. kg^−1^. After endotracheal intubation anesthesia maintenance in the S group was 0.5–1 MAC sevoflurane while in the P group was propofol 100–200 μg.kg^−1^.min^−1^ to keep BIS level at 45–55 throughout the procedure, and both groups received rocuronium 0.5 mg. Kg^−1^.hr^−1^ and sufentanil 0.2–0.4 μg.kg^−1^. hr^−1^ infusion. Volume-controlled ventilation was set up to maintain normoxia (PaO_2_ = 200–300 mmHg) and normocapnia. A 7Fr. triple vena cava catheter was placed through the right subclavian for infusing norepinephrine, epinephrine, and nitroglycerin respectively. A 5-line Swan-Ganz catheter was placed through right internal jugular vein for central venous pressure (CVP), cardiac index (CI) and body temperature observation. A cell saver was used to minimize allogenic red blood cell (RBC) transfusion, and packed RBC were transfused according to a transfusion haematocrit trigger of 25% during surgery. Haemodynamic management with inotropic and vasodilator therapy aimed to maintain mART at 65–90 mmHg, CVP at 6-15 mmHg according to the different periods of manipulating of the heart, and keep CI above 2.5 l. min^−1^. m^−2^.

Surgical technique was standardized and all procedures were performed by the same team. When the left internal mammary artery (LIMA) was prepared heparin dosage of 150 IU kg^−1^ was administrated to reach activated clotting time (ACT) > 350 s. The left internal mammary artery was attached to the left anterior descending artery coronary with artery shunt (Clearview; Medtronic, Minneapolis, MN, USA) was used during anastomosis, and vein grafts from great saphenous vein were used for other grafts as appropriate. Operative region immobilization was achieved with the help of a mechanical stabilizer (Octopus 3; Medtronic, Minneapolis, MN, USA). The near- top-end anastomoses were constructed with the help of an anastomosis assist device (eNclose®II; Vitalitec International, Inc. Plymouth, MA, USA). When the graft procedure finished, i.v. protamine sulphate was administered to obtain ACT < 140 s. After surgery, all patients were transferred to the ICU and received the same routine postoperative care. Briefly, patients received sufentanil 2–3μg. hr^−1^ for postoperative analgesia by postoperative analgesia pump at discretion; Tracheal extubation was performed when patients met the criteria as follows: normal consciousness, tidal volumn(V_T_) not less than 6 ml. kg^−1^, oxygenation index greater than 200 mmHg and partial pressure of carbon dioxide (PCO_2_) less than 45 mmHg while the fraction of inspired oxygen (FiO_2_) was no more than 0.5. haemodynamic stability, normal cardiac output as well as absence of malignant arrhythmias. The patients were discharged from the ICU when they met these criteria: haemodynamic stability without infusion of vasoactive or inotropic drugs, SpO_2_ maintained at 95% or higher with supplemental oxygen no more than 3 l.min^−1^ by nasal oxygen catheter. chest tube drainage less than 25 ml.h^−1^ and urine volume greater than 0.5 ml.kg^−1^.h^−1^.

### Blood sampling

Blood samples were collected in 6 timepoints: induction (T_1_), Before grafting (T_2_), after grafting(T_3_), surgery done (T_4_), POD1 (T_5_) and POD2(T_6_). Blood sample was processed within 2 h when it was collected, which was centrifuged and the serum were stored at –80 °C for later test. The concentration of ANP, of the glycocalyx components syndecan-1 and HS (RayBiotech, Georgia, USA), of the inflammatory markers IL-6 (R&D Systems, Minnesota, USA) were detected by enzyme linked immunosorbent assay (ELISA) from T_1_ to T_6_. Serum albumin, cTnI and Hb levels were also examined by the inpatient laboratory tests at the day before surgery (T_0_), T_5_ and T_6_. Starting on the first postoperative day, the SOFA score was computed daily until ICU discharge or for a maximum of 7 days according to the modalities described by Flavio Lopes Ferreira et al. [25]. SIRS classification was performed postoperatively daily for 5 days which was diagnosed according to the criteria proposed by the American College of Chest Physicians/Society of Critical Care Medicine Consensus Conference, which was defined as a condition that included two or more of the following factors: (1) body temperature < 35.6 °C or > 38.3 °C; (2) heart rate > 90 beats.min^−1^; (3) respiratory rate > 20 breaths. min^−1^ or PaCO_2_ < 4.27 kPa; (4) white blood cell count ≥ 1.2 × 10^6^. ml^−1^or ≤ 0.4 × 10^6^. ml^−1^or > 10% immature (band) forms.

### Statistical analysis

Statistical analysis was performed using SPSS 22 and Graphpad Prism 9. Results for continuous data are expressed as means and standard deviation or median and quartiles for normal and skewed distributions, respectively. Group comparisons of numerical data were performed by Student’s t-test, Mann–Whitney test or Kruskal–Wallis test as appropriate. Comparisons between categorical data were performed by chi-square test. An analysis of variance for repeated measures was used to analyze differences among group means. Heatmaps are used to display the *P* value of Spearman correlation tests between biochemical and evaluative indicators. P value less than 0.05 was considered statistically significant.

## Results

**Fig. 1:**
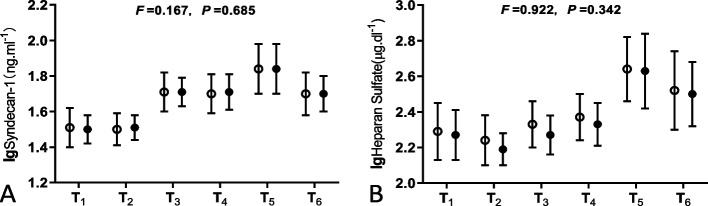
Analysis of variance for repeated measures of logarithmic value of serum syndecan-1 (**A**) or serum HS (**B**) between propofol group (○) and sevoflurane group (●). Dots are mean and whiskers are SD. There is no significant difference between the two groups. T_1_, induction;T_2_, before grafting; T_3_, after grafting; T_4_, surgery done; T_5_, postoperative day1; T_6_, postoperative day2

Totally 50 enrolled patients went through operation successfully. In each group there were two patients excluded due to the severe hemolysis of the blood sample, so finally both groups had 23 patients included in the final analysis, which met the requirements of statistical design. All procedures of coronary graft anastomosis were performed by the same surgeon. Baseline characteristics and peri-operative situations of the patients are shown in Tables 1 and 2. Serum concentration of syndecan-1 and HS were not normally distribution, after logarithmic transformation to meet normal distribution there were no differences of syndecan-1 or HS between the propofol and Sevoflurane groups in the analysis of variance for repeated measures (Fig. 1). There were no differences in postoperative serum concentration of cTnI (POcTnI), SIRS or the maximum SOFA score between the propofol and Sevoflurane groups either (Table 3). Thus, the two groups of patients were pooled together for further statistical analyses. Serum concentration of cytokine and ANP did not show normal distribution even after logarithmic transformed.

**Table 1:** Baseline characteristics of patients receiving sevoflurane or propofol anesthesia during OPCABG. Values are number, number (proportion), mean (SD), or median (IQR [range])

	**Propofol group** **(*n* = 23)**	**Sevo group** **(*n* = 23)**	***P* value**
Gender(M/F)	14/9	16/7	0.758
Age(years)	62.6(4.9)	63.3(8.5)	0.719
Weight(kg)	67.5(14.0)	71.8(10.3)	0.246
Height(cm)	165(7.71)	165.43(6.83)	0.856
LVEF; 35–49%/ > 49%	6/17	4/19	0.722
1VD/2VD/3VD	2/6/15	1/8/14	0.719
Duration of Operation(hours)	4.19(0.75)	4.22(0.58)	0.861
Duration of Grafts Anastomosis(min)	90 (70–120[50–180])	90 (75–110[65–155])	0.799

*LVEF* Left ventricle ejection fraction, *VD* Vessel disease

**Table 2:** Fluid balance of patients receiving sevoflurane or propofol anesthesia during OPCABG. Values are median (IQR [range]) or number (proportion)

	**Propofol group** **(n = 23)**	**Sevo group** **(n = 23)**	***P* value**
Intra-operative parameters			
blood loss (ml. kg^−1^)	10.4(9.3–13.3[5.2–29.0])	9.7(8.8–12.5[4.9–27.3])	0.804
crystal fluid infused (ml.kg^−1^)	31.1(25.2–32.6[17.8–41.5])	29.2(23.7–30.6[16.7–39.0])	0.972
colloid fluid infused (ml. kg^−1^)	22.2(14.8–29.6[14.8–37.0])	20.9(13.9–27.9[13.9–34.8])	0.155
Cell-saver RBC infused (ml)	250(250–500[0–1000])	250(250–500[0–1200])	0.848
Receiving FFP (case)	6(26.1%)	8(34.8%)	0.522
Receiving allogeneic RBC (case)	4(17.4%)	5(21.7%)	0.710
urine volume (ml)	800(500–1200[350–2000])	700(600–800[300–1400])	0.094
Postoperative parameters			
POD1			
crystal fluid infused (ml. kg^−1^)	29.2(26.2–29.6[23.6–37.0])	27.4(24.7–27.9[22.1–34.8])	0.878
colloid fluid infused (ml. kg^−1^)	14.4(8.9–16.3[8.9–29.6])	13.5(8.4–15.3[8.4–27.9])	0.666
urine volume (ml)	3445(2830–4000[1020–5530])	3330(2910–3920[2000–4460])	0.764
chest tube drainage (ml)	230(200–300[100–730])	330(210–530[130–740])	0.074
POD2			
crystal fluid infused (ml. kg^−1^)	27.1(26.2–29.4[16.8–40.0])	25.5(24.6–27.6[15.8–37.6])	0.860
colloid fluid infused (ml. kg^−1^)	8.9(8.9–12.9[3.0–23.7])	8.4(8.4–12.2[2.8–22.3])	0.372
urine volume (ml)	3420(2860–3990[1850–4730])	3460(2970–3780[1440–4940])	0.802
chest tube drainage (ml)	170(50–225[5–390])	150(90–240[20–490])	0.558

*RBC* Red blood cells, *FFP* Fresh frozen plasma, *POD* Postoperative day

**Table 3:** Postoperative outcome characteristics of patients receiving sevoflurane or propofol anesthesia during OPCABG. Values are number, number (proportion) or median (IQR [range])

	**Propofol group** **(*n* = 23)**	**Sevo group** **(*n* = 23)**	***P* value**
PO cTnI (ng.ml^−1^)	2.4(0.81–8.00[0.22–169.00])	1.25(0.53–9.60[0.09–110.50])	0.455
PO Ventilation; ≤ 1 day/ ≤ 2 days/ ≥ 3 days	19/1/3	20/1/2	0.893
PO atrial fibrillation	3(13%)	5(21.7%)	0.699
PO SIRS (days)	1(0–2[0–5])	0(0–1[0–4])	0.337
PO SOFAmax	6(5–7[2–8])	6(5–7[2–8])	0.676
PO ICU stay (days)	4(3–6[3–29])	4(3–6[2–10])	0.591
PO Hospital stay (days)	9(8–12[7–40])	11(9–14[7–20])	0.134

*PO* Postoperative, *SOFAmax* Maximum value of SOFA score

**Fig. 2:**
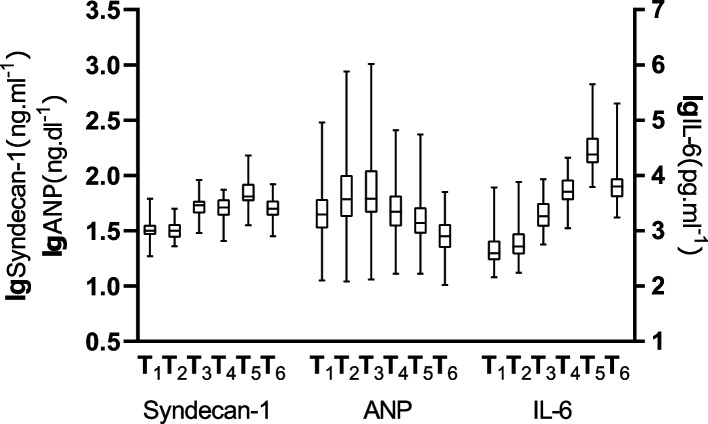
The Kruskal–Wallis test of variations of logarithmic value of serum syndecan-1(*P* < 0.001), ANP (*P* < 0.001) and IL-6 (*P* < 0.001). Horizontal lines are median, boxes are interquartile range and whiskers are range. Figure shows the consistency of intraoperative trends in syndecna-1 and ANP, and the consistency of postoperative trends in syndecna-1 and IL-6. T_1_, induction; T_2_, before grafting; T_3_, after grafting; T_4_, surgery done; T_5_, postoperative day1; T_6_, postoperative day2

**Fig. 3:**
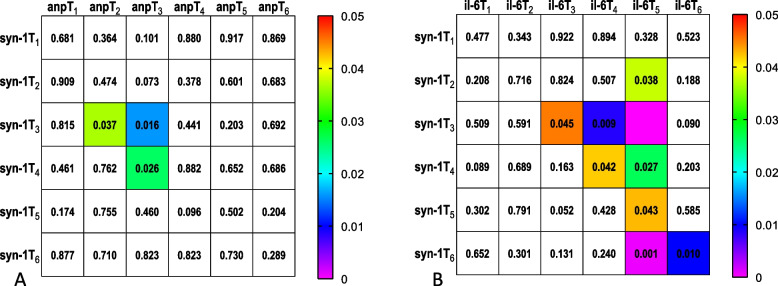
*P* values of correlation show weak or moderate correlations of ANP and syndecan-1 (**A**) or IL-6 and syndecan-1 (**B**). **syn-1**, logarithmic value of serum concentration of syndecan-1; ANP, logarithmic value of serum concentration of ANP; il-6, logarithmic value of serum concentration of IL-6; T_1_, induction; T_2_, before grafting; T_3_, after grafting; T_4_, surgery done; T_5_, postoperative day1; T_6_, postoperative day2

**Fig. 4:**
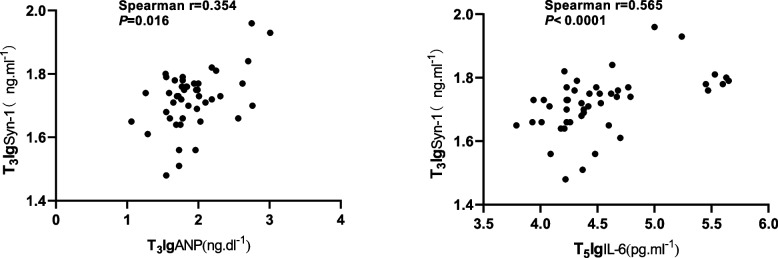
Correlation of T_3_syndecan-1 and T_3_ANP (**A**) or T_3_syndecan-1 and T_5_IL-6 (**B**). T_3_, the time when grafts procedure finished; T_5_, postoperative day1; lgsyn-1, logarithmic value of serum concentration of syndecan-1; lgIL-6, logarithmic value of serum concentration of IL-6

When all the 46 patients were included in one group, serum concentrations of ANP (*P* < 0.001), syndecan-1 (*P* < 0.001), HS (*P* < 0.001) and IL-6 (*P* < 0.001) increased significantly intraoperatively or postoperatively; The intraoperative increasing value peak of serum concentrations of syndecan-1 occurred at T_3_, and the postoperative peak was at T_5_; The significant increasing of serum concentrations of ANP begun at T_2_ and reached its peak at T_3_; The increasing peak of serum concentrations of IL-6 was at T_5 _(Fig. 2). Preoperative serum concentrations of syndecan-1 and HS correlated significantly with the corresponding concentrations at all later time points. Plasma syndecan-1 correlated significantly with ANP at T_2_ and T_3_, with IL-6 at T_3_ to T_6_, these correlations were strongest between syndecan-1T_3_ and ANPT_3_(Spearman r = 0.354, *P* = 0.016), between syndecan-1T_3_ and IL-6 T_5_(Spearman r = 0.565, *P* < 0.0001) (Figs. 3, 4).

**Fig. 5:**
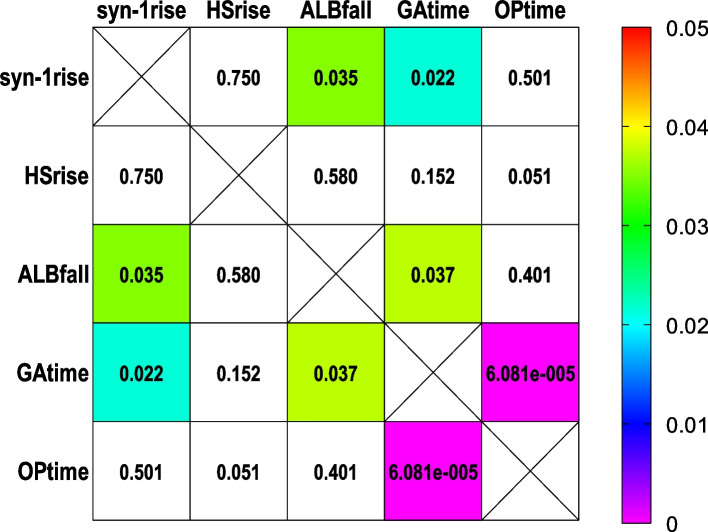
*P* values of correlation show weak or moderate correlations of syndecan-1rise, ALBfall, GAtime. **syn-1rise**, increasing logarithmic value of serum concentration of syndecan-1; **HSrise**, increasing logarithmic value of serum concentration of HS; ALBfall, decreasing value of serum concentration of albumin; GAtime, graft anastomosis time; OPtime, operation time

**Fig. 6:**
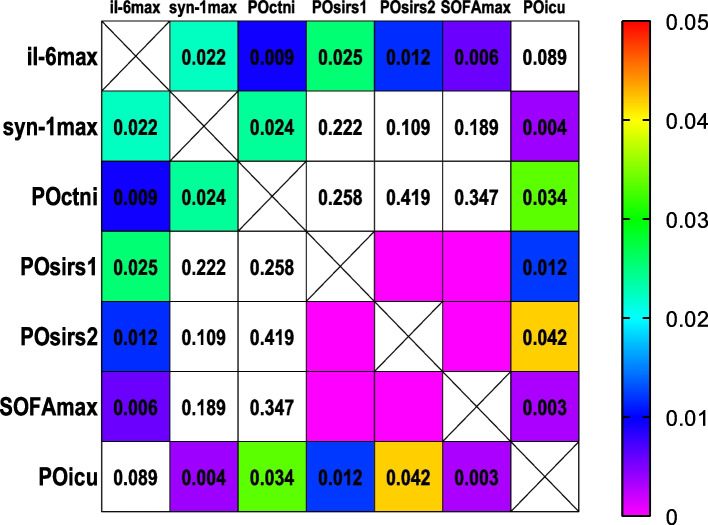
*P* values of correlation show weak or moderate correlations of IL-6、syndecan-1 and postoperative indicators. il-6max, the maximum logarithmic value of serum concentration of IL-6; syn-1max, the maximum logarithmic value of serum concentration of syndecan-1; POctni, the postoperative maximum value of serum concentration of cTnI; POsirs1, the postoperative occurance of SIRS; POsirs2, the duration days of postoperative SIRS; SOFAmax, the postoperative highest SOFA score; POicu, days of postoperative ICU stay

The maximum value of IL-6 (IL-6max) correlated significantly with the maximum value of syndecan-1 (syn-1max) (Spearman r = 0.336, *P* = 0.022) and HS (HSmax) (Spearman r = 0.322, *P* = 0.029). Not operation time but anastomosis time correlated significantly with perioperative syndecan-1 increasing and serum albumin drop. Syndecan-1 increasing correlated significantly with serum albumin drop (Fig. 5). Occurrences and duration days of SIRS correlated significantly with SOFA score; Occurrences of SIRS, the maximum value of SOFA score (SOFAmax), POcTnI all correlated significantly with postoperative ICU days (POICU). The maximum value of IL-6 (IL-6max) correlated significantly with occurrences and duration of SIRS, postoperative SOFAmax and POICU. POICU correlated significantly with a postoperative maximum value of POcTnI. POcTnI correlated significantly with the syn-1max and the IL-6max. occurrences and lasting days of SIRS correlated significantly with SOFAmax score; Syn-1max, occurrences of SIRS, SOFAmax, POcTnI all correlated significantly with POICU. Syn-1max, occurrence of SIRS, SOFAmax, POcTnI all correlated significantly with IL-6max (Fig. 6).

## Discussion

SIRS is a nonspecific clinical state which can be precipitated by a wide range of insults including cardiac surgery. Recent data showed that SIRS is associated with a 13-fold increase in mortality that can be as high as 4.8% in patients undergoing some kinds of elective cardiac-vascular surgery [3, 6]. Although OPCABG avoids the proinflammatory factors such as aortic clamping, CPB, and to a large extent I/R, the incidence of postoperative SIRS in OPCABG patients is still higher than in other kinds of surgeries. We know that EGL plays a regulatory role in the inflammatory response, while inflammation reaction will cause EGL shedding. EGL shedding accompanies the spread and exposure of cytokines and enzymes in the EGL,while EGL shedding can be an important source of DAMPs, such as syndecan-1, hyaluronan, HS can all act as DAMPs [26]. Early observations found the same degradation of EGL in off-pump or on-pump cardiac surgeries, which result in the same extent of perioperative systemic inflammation [15, 27, 28]. By contrast, other kinds of surgeries would not experience obvious intraoperative EGL shedding which indicated by the significant increase in plasma syndecan-1 concentration [29].

In OPCABG procedure, cardiac manipulation especially for working on the posterior or lateral walls, heart must be tilted and moved out of the pericardial cavity. When the heart be tilted to vertical position with the apex at its zenith, the atria are then situated below the corresponding ventricles and the blood must flow up into the ventricular cavities with a higher filling pressure in the atria, and what’s more moving and probing of the heart, applying of the stabilizer device etc. will together have a strong impact on the pressure of heart wall. This will give rise to massive ANP secretion and release which can cause intraoperative early EGL degradation in terms of plasma syndecan-1 rising (Fig. 1) [30, 31]. In our OPCABG surgery syndecan-1 increased significantly intraoperatively and correlated significantly with ANP (Spearman *r* = 0.346, *P* = 0.016) and IL-6(Spearman *r* = 0.297, *P* = 0.045) at T_3_ (Figs. 2, 3), indicating correlation of ANP releasing and EGL degradation in OPCABG intraoperatively.

We also found that not operation time but anastomosis time correlated significantly with perioperative syndecan-1 increasing and plasma albumin decreasing (Fig. 5). EGL plays an important role in maintaining the integrity of microcirculation structure and function including avoidance of albumin leakage [32]. All in all it indicates that graft anastomosis was the main cause of ANP releasing and intraoperative EGL degradation. Further observations revealed that the intraoperative peak value of plasma syndecan-1 concentration(T3) correlated most significantly with the serum concentration of IL-6 (Spearman *r* = 0.565, *P* < 0.0001) of POD1(T5). IL-6 is a dominant inflammatory cytokine in the postoperative period and the serum concentration of IL-6 usually starts to rise significantly several hours after inflammatory factors take effect, this implies intraoperative EGL degradation may have remarkable influence on postoperative system inflammation in terms of a cytokine such as IL-6 [6, 33, 34]. As well, we also found that IL-6max correlated significantly with syn-1max and HSmax. In brief, in OPCABG it is coronary graft anastomosis mainly causes the cardiac mass releasing of ANP and early EGL shedding, and the DAMPs production is an important factor that inducing postoperative systemic inflammation response.

We know that there is concurrent pro-inflammatory and immunosuppressive activity throughout the postoperative period, and the degree of systemic inflammation and immunosuppression is related to the initial inflammatory cytokine spike, and this postoperative state is closely related to the patient’s prognosis [6]. Thus, as mentioned above, in OPCABG the characteristic intraoperative EGL damage and shedding correlated significantly with postoperative inflammation state in terms of IL-6, myocardial injury marker cTnI and POICU (Fig. 6). On the other hand, in our observation IL-6 as a whole reached its peak value in POD1 and IL-6max correlated significantly with POcTnI, occurrences and duration of SIRS, postoperative SOFAmax and POICU. There are extremely different results of EGL degradation in various kinds of surgeries, but it is a consensus that high level of syndecan-1, a marker of endothelial glycocalyx degradation is associated with worse outcomes. It seems that the concentration of serum soluble syndecan-1 above 30 ng.ml^−1^ or 40 ng.ml^−1^ may associated with inflammation, coagulopathy and increased mortality, which identified patients with worse outcome [35–37]. Then as a verification of this in our observation the occurrences of SIRS, SOFAmax, POcTnI all correlated significantly with POICU (Fig. 6).

### Limitations of the study

The study had several limitations. Firstly, syndecan-1 was only a surrogate indicator for the shedding of EGL and cannot distinguish degradation between different organs, but ANP may have a different effect on different organ systems [38]. Secondly, we routinely applied ulinastatin during the surgery and this may affect the state of inflammatory response. Thirdly, the sample size is insufficient to evaluate postoperative clinical efficacy. Finally, it must be emphasized that due to trivial increase in macromolecular substances and the weak correlations of related factors, more experiments are needed to verify the results of this study, and these findings may be helpful for future multi-center studies with larger sample sizes. To be gratified, our subsequent clinical trials are ongoing.

## Conclusions

We found that in OPCABG surgery the characteristic mass ANP releasing caused by grafts anastomosis contributes to early intraoperative EGL degradation, which may affect the postoperative inflammatory response in terms of cytokines such as IL-6. This will further influence on the occurrence of postoperative SIRS and SOFA score values, which related to prolonged postoperative ICU time in OPCABG. Our observation found no perioperative difference in the occurrence of postoperative SIRS and SOFA score value between sevoflurane or propofol groups in OPCABG. Despite the limitations of the results of this experiment, we still recommend that in OPCABG shortening of grafts anastomosis time, reduction of compression and movement of the heart during operation can attenuate the degradation of EGL, which may improve postoperative inflammation state.

## Data Availability

The original data are available from the corresponding author on request.

## Abbreviations

ACT: Activated clotting time
ANP: Atrial natriuretic peptide
ART: Artery pressure
BIS: Bispectral index
CI: Cardiac index
cTnI: Cardiac troponin I
CVP: Central venous pressure
DAMPs: Damage-associated molecular patterns
EGL: Endothelial Glycocalyx Layer
ELISA: Enzyme-linked immunosorbent assay
Hb: Blood haemoglobin
HS: Heparan sulfate
LIMA: Left internal mammary artery
OPCABG: Off-Pump coronary artery bypass graft
PO: Postoperative
POD: Postoperative day
RBC: Red blood cell
SD: Standard deviation
SIRS: Systemic inflammation response syndrome
SpO2: Saturation of pulse oximetry
SOFA: Sequential organ failure assessment
syn-1: Syndecan-1
